# Clinicopathological characteristics of alveolar adenoma

**DOI:** 10.3389/fonc.2026.1832957

**Published:** 2026-06-02

**Authors:** Jing Chen, Zhengjin Liu, Wei Zheng, Huiling Chen, Zeyang Lin, Zhe Li

**Affiliations:** 1 Department of Pathology, Zhongshan Hospital of Xiamen University, School of Medicine, Xiamen University, Xiamen, China; 2 Department of Pathology, The People’s Hospital of Guangxi Zhuang Autonomous Region (Guangxi Academy of Medical Sciences), Nanning, China; 3 Department of Thoracic Surgery, Zhongshan Hospital of Xiamen University, School of Medicine, Xiamen University, Xiamen, China

**Keywords:** alveolar adenoma, CD10, CD34, diagnosis, differential diagnosis

## Abstract

**Introduction:**

Alveolar adenoma is an uncommon benign lung tumor that typically appears as a well-defined nodule on imaging and pathological examination. Currently, there is limited research on alveolar adenoma. This article aims to present this rare tumor by describing three cases.

**Methods:**

We reviewed and analyzed the morphological and immunohistochemical characteristics of three cases using hematoxylin and eosin (HE) staining and immunohistochemistry (IHC).

**Results:**

The alveolar adenomas exhibited well-defined tumors composed of well-differentiated cuboidal or flattened alveolar epithelial cells that formed glandular or cystic structures of varying sizes, accompanied by well-differentiated spindle cell stroma. Immunohistochemical analysis showed consistently strong and widespread CD34 expression in the stromal cells in all cases. Additionally, CD10 was diffusely positive in stromal cells in about two-thirds of the cases, with focal positivity observed in the remaining case.

**Discussion:**

While previous research has not identified specific immunohistochemical markers, our findings are interesting, and we suggest that these markers could be useful in diagnosing alveolar adenoma.

## Introduction

Alveolar adenoma is an uncommon benign lung tumor characterized by the growth of alveolar epithelial and mesenchymal cells (1). It was first identified in 1986 (2). Typically, it affects individuals between 40 and 60 years old, with a higher incidence in females (3). Most patients do not show symptoms, though some have reported cough, chest pain, and shortness of breath. The tumor most frequently occurs in the left lower lobe of the lung and ranges in size from 0.2 to 9.1 cm (4). Alveolar adenoma is non-cancerous, and surgical removal is curative. While its exact origin remains uncertain, the majority of research indicates it arises from type II pneumocytes (5). But we know that the tumor is composed of epithelial and stromal components, and the stromal component expresses CD34 and CD10 in our series. We speculate that the stromal component is also part of the tumor. However, this is only a hypothesis based on the positivity of CD34 and CD10 in the stromal component. This is because, on one hand, the number of cases is limited, and on the other hand, we have not conducted molecular testing on the stromal component, so we cannot determine whether there are driver gene alterations or monoclonal proliferation in the stromal component. Therefore, in the future, we need to collect more cases and perform molecular testing on the stromal component to further explore the nature of this tumor.

## Materials and methods

The three cases were fixed in 3.7% neutral-buffered formalin, dehydrated, and embedded in paraffin, then cut into 4 μm serial sections. These sections underwent hematoxylin and eosin (HE) staining and immunohistochemical staining for pan-CK, CK7, TTF-1, Napsin A, EMA, CD10, CK20, P40, CK5/6, Vimentin, CD34, Bcl-2, S-100, SMA, CEA, SYN, CD56 and Ki-67. All antibodies were sourced from Fuzhou Maixin Biotech Co., Ltd.

For immunohistochemical interpretation, we use a dual-dimensional scoring system: “staining intensity score × positive cell proportion score,” detailed as follows: Staining intensity (0–3 points): no positive staining (0 points), light yellow (weak positive, 1 point), brownish yellow (positive, 2 points), dark brown (strong positive, 3 points). Positive cell proportion (1–4 points): ≤25% (1 point), 26%-50% (2 points), 51%-75% (3 points), >75% (4 points). Final score = staining intensity score × positive cell proportion score.

## Result

### Case 1

The patient was a 31-year-old man who showed no symptoms. A computed tomography scan identified solid nodules in the posterior upper lobe of the right lung. No signs of recurrence or metastasis were detected during the 18-month follow-up period after surgery (**Table 1**).

**Table 1:** Clinical characteristics of alveolar adenoma.

Case	Age(y)/sex	CT	Size(mm)	Symptom	Treatment	Metastasis/recrudescence	Follow up(M)
Case 1	31/M	Well-circumscribed solid nodule	18	No symptoms	Surgery	No	18
Case 2	74/F	Well-circumscribed thin-walled cystic nodule.	16	No symptoms	Surgery	No	19
Case 3	35/F	Well-circumscribed partially solid nodule.	14	No symptoms	Surgery	No	8

**Figure 1:**
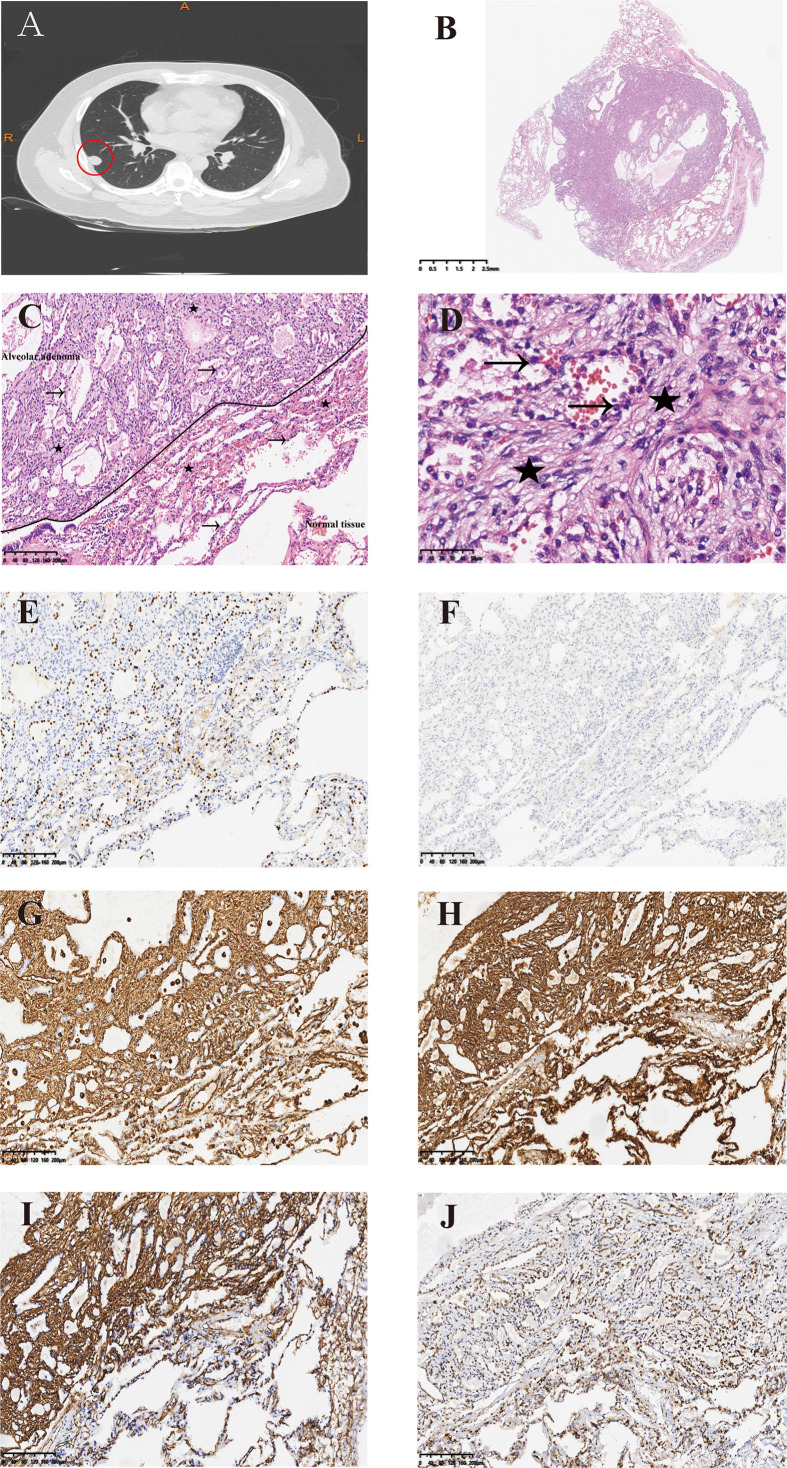
**(A)** CT scan showing a solid nodule with clear borders in the posterior segment of the right upper lobe. **(B)** Hematoxylin and eosin staining reveals a tumor with a well-defined margin at ultra-low magnification (10x). **(C)** The boundary between tumor tissue and normal tissue is clear. The tumor tissue contains well-differentiated glands (arrows) visible within the tumor, accompanied by proliferation of surrounding stromal cells (asterisks) (100x). **(D)** Both epithelial(arrows)and stromal cells (asterisks) appear well-differentiated without significant atypia; some glands contain small amounts of eosinophilic secretion (400x). **(E)** Expression of TTF-1 (100x). **(F)** Expression of P40 (100x). **(G)** Expression of vimentin (100x). **(H)** Expression of CD10 (100x). **(I)** Expression of CD34 (100x). **(J)** Expression of BCL-2 (100x).

The tumor was well-defined against the surrounding lung tissue, measuring 18 by 15 mm (**Figure 1A**). Pathological examination revealed a cystic-solid mass (**Figure 1B**) made up of proliferating epithelial and stromal cells (**Figure 1C**). Both cell types exhibited mild atypia, with well-differentiated glandular lumens containing a small amount of eosinophilic secretions, and the stroma showed myxoid degeneration (**Figure 1D**). Immunohistochemical analysis demonstrated that the epithelial cells tested positive for TTF-1 (**Figure 1E**), pan-CK, CK7, Napsin A, EMA, CD10 (**Figure 1H**), and focal positive for Bcl-2 (**Figure 1J**), while negative for CK20, P40 (**Figure 1F**), and CK5/6. The stromal cells were positive for Vimentin (**Figure 1G**), CD10 (**Figure 1H**), CD34 (**Figure 1I**), and focal positive for Bcl-2(**Figure 1J**), but negative for S-100 and desmin. Ki-67 was low.

### Case 2

**Figure 2:**
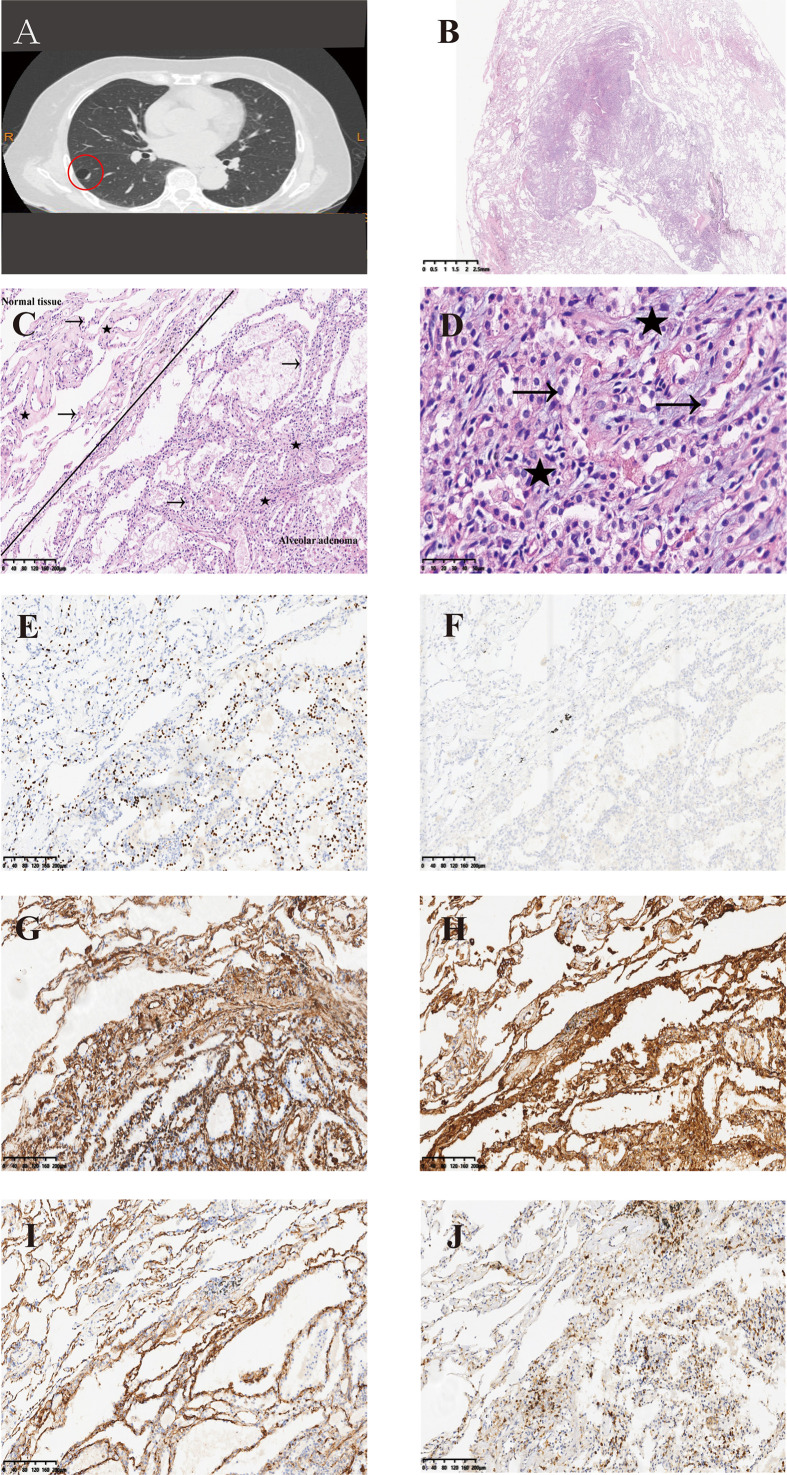
**(A)** CT scan displays a well-circumscribed thin-walled cystic nodule. **(B)** Low magnification shows the tumor with a clear boundary (10x). **(C)** The boundary between tumor tissue and normal tissue is clear. The tumor tissue contains well-differentiated glands (arrows), some of which are cystic, containing eosinophilic secretions, the stroma (asterisks) is loose with spindle-shaped cells exhibiting mild morphology within a myxoid matrix, along with a small number of inflammatory cells (100x). **(D)**Both epithelial (arrows) and stromal cells (asterisks) appear mild in morphology without notable atypia (400x). **(E)** Expression of TTF-1 (100x). **(F)** Expression of P40 (100x). **(G)** Expression of Vimentin (100x). **(H)** Expression of CD10 (100x). **(I)** Expression of CD34 (100x). **(J)** Expression of BCL-2 (100x).

The patient was a 74-year-old woman without any symptoms. A computed tomography scan identified a thin-walled cystic nodule approximately 16 mm in diameter located in the dorsal segment of the right lower lung lobe (**Figure 2A**). No signs of recurrence or metastasis were detected during the 19-month follow-up period after surgical treatment (**Table 1**).

From a morphological perspective, the lesion was fairly well-defined and distinct from the surrounding lung tissue (**Figure 2B**). It consisted of cystically dilated glands, well-differentiated glands, and proliferating stromal cells. The glandular lumens contained eosinophilic secretions, and there was localized myxoid degeneration within the stroma (**Figure 2C**). Neither the epithelial nor stromal cells showed significant atypical features (**Figure 2D**).

Immunohistochemical analysis revealed that the epithelial cells tested positive for TTF-1 (**Figure 2E**), CK-pan, CK7, Napsin A, EMA, CD10 (**Figure 2H**), and focal positive for Bcl-2 (**Figure 1J**), while they were negative for P40 (**Figure 2F**) and CK5/6. The stromal cells were positive for Vimentin (**Figure 2G**), CD10 (**Figure 2H**), CD34 (**Figure 2I**), and focal positive for Bcl-2(**Figure 2J**), with partial positivity for SMA. Ki-67 was low.

### Case 3

**Figure 3:**
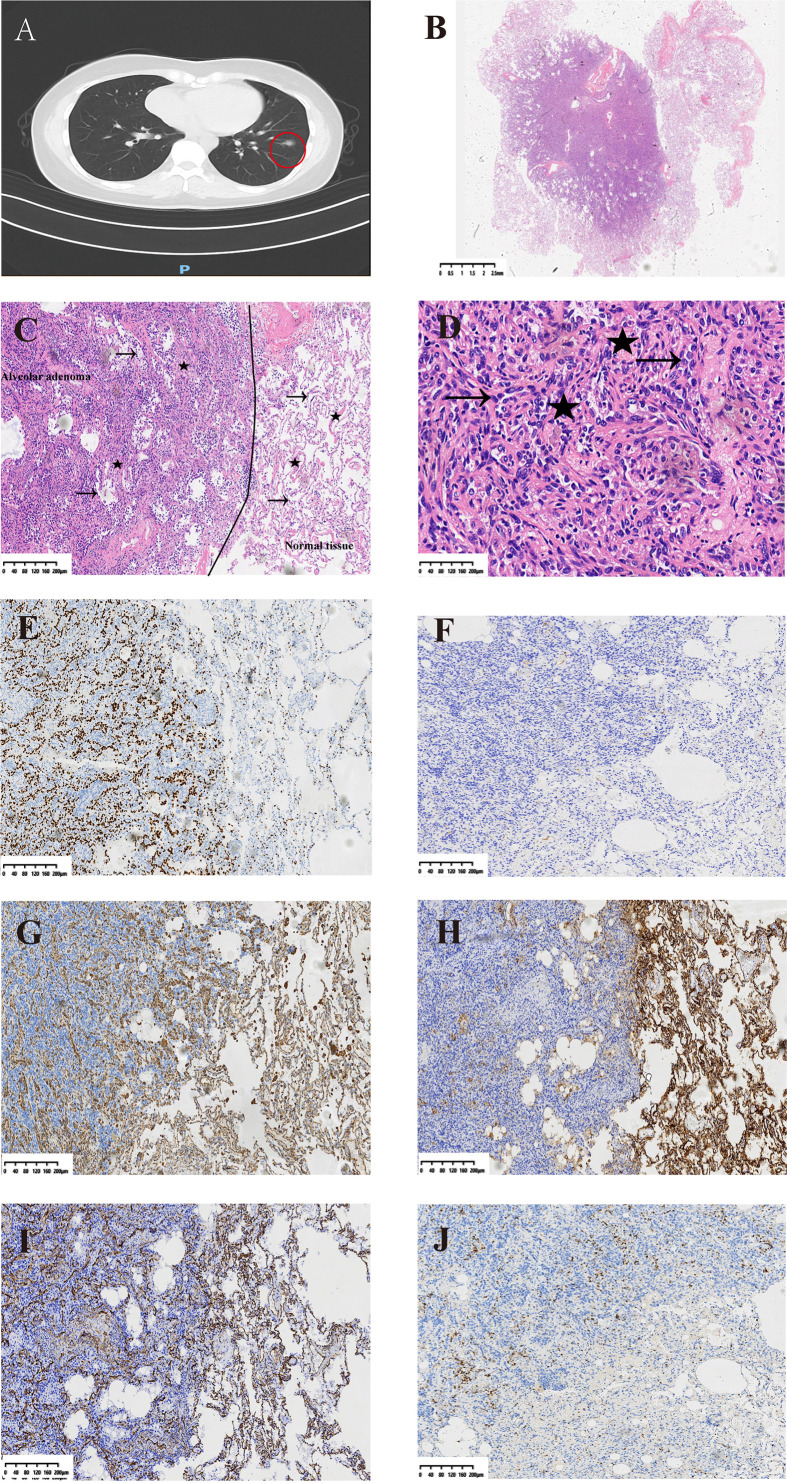
**(A)** CT scan showing a well-defined solid nodule; **(B)** The tumor is distinctly separated from adjacent lung tissue (10x). **(C)** The tumor is made up of densely packed glandular epithelium (arrows), partially arranged in fissure-like patterns, surrounded by proliferating stromal cells (asterisks) (100x). **(D)** Most epithelial cells (arrows) are arranged in a single layer, with no significant atypia in either epithelial or stromal cells (asterisks) (400x). **(E)** Expression of TTF-1 (100x); **(F)** Expression of P40 (100x). **(G)** Expression of vimentin (100x); **(H)** Expression of CD10 (100x); **(I)** Expression of CD34 (100x). **(J)** Expression of BCL-2 (100x).

The patient was a 35-year-old woman who is asymptomatic. Three-dimensional reconstructed spiral CT images revealed a part-solid nodule measuring approximately 14mm by 9mm (**Figure 3A**). No recurrence or metastasis was detected during the 8-month follow-up period after surgical treatment (**Table 1**).

Morphologically, the lesion is clearly separated from the surrounding tissue (**Figure 3B**). It consists of proliferating epithelial and stromal cells, without obvious cystic structures (**Figure 3C**). In certain areas, epithelial proliferation is more pronounced than stromal growth; in some regions, epithelial cells are compressed into slit-like spaces. There are no eosinophilic secretions within the glandular lumina, and no myxoid degeneration is observed in the stroma. Neither epithelial nor stromal cells exhibit significant atypia (**Figure 3D**).

Immunohistochemical analysis shows that the epithelial cells test positive for TTF-1 (**Figure 3E**), Napsin A, CK7, and EMA; they are focal positive for CD10 (**Figure 3H**) and focal positive for Bcl-2(**Figure 3J**), while negative for P40 (**Figure 3F**), CEA, SYN, and CD56. The stromal cells are positive for Vimentin (**Figure 3G**), CD10 (**Figure 3H**), CD34 (**Figure 3I**) and focal positive for Bcl-2(**Figure 3J**). Ki-67 was low.

The clinical and immunohistochemical characteristics of the three cases are summarized in **Tables 1** and **2**, respectively. The immunohistochemical expression is quite interesting. Except for the epithelial component in case 3, where CD10 is focally positive, CD34 and CD10 are continuously expressed in the stromal component rather than in the epithelial component.

**Table 2:** Immunohistochemical characteristics of alveolar adenoma.

Case	Tumor element	CK7	TTF-1	Napin A	EMA	Vimentin	CD10	CD34	BCL-2
Case 1	Epithelial cells	12	12	8	8	0	8	0	6
	Stromal cells	0	0	0	0	12	12	12	1
Case 2	Epithelial cells	12	12	8	8	0	8	0	1
	Stromal cells	0	0	0	0	12	12	12	1
Case 3	Epithelial cells	12	12	8	8	0	2	0	1
	Stromal cells	0	0	0	0	12	4	8	1

## Discussion

Classical features: alveolar adenoma typically appears on imaging as a well-defined solitary nodule (6). Diagnosing alveolar adenomas is generally straightforward using routine pathology sections. The characteristic morphology includes a clearly outlined lesion made up of cystic spaces of various sizes, lined by flattened to cuboidal epithelial cells, without significant variability. The lumen of these cysts may contain eosinophilic or clear secretions, and there is an absence of nuclear division and necrosis (3, 7). The supporting tissue features spindle-shaped cells accompanied by inflammatory cell infiltration and a mucus-like stroma (1). Alveolar adenomas are generally considered to have a favorable prognosis and do not recur after surgical removal, although there has been one reported case of progression to adenocarcinoma (8).

Atypical findings: in our series of three cases, one displayed atypical morphological characteristic compared to typical alveolar adenoma: it lacked a clear cystic structure, had only rare eosinophilic secretions in the lumens, showed epithelial proliferation that significantly exceeded the stromal component, and exhibited minimal myxoid degeneration in the stroma. Despite these differences, the diagnosis of alveolar adenoma was still confirmed.

IHC: epithelial cells commonly express markers such as pan-CK, CK7, CK18, CK19, EMA, TTF-1, and surfactant apoproteins B and C, whereas mesenchymal cells do not. Mesenchymal cells express Vimentin, variably express SMA, and rarely express S100 and CD34 (3). Due to the limited number of reported cases, immunohistochemical expression, especially in interstitial cells, varies. In our three cases, stromal cells showed diffuse positive expression of both CD10 and CD34, and focal positive for Bcl-2 as detailed in **Table 2**.

Interpretation: CD10 is commonly found in stem cells and is regarded as a stem cell marker (9, 10), however, it is also abundant in the lung, where it plays a key role in cell differentiation, proliferation, and tumor development (11). CD10 expression in the epithelial cells of alveolar adenoma has not been previously documented in the literature. In our study, two cases showed diffuse strong positive expression in epithelial cells, while one case exhibited focal positive expression. From the literature, we know that CD10 is associated with cell stemness and is related to various tumors. In our three reported cases, CD10 was positive in all, with two cases showing diffuse strong positivity. We have reason to speculate that the stromal component of alveolar adenoma is also a tumor component. There are two reasons for this: first, if it were not a tumor component, CD10 should show patchy positivity rather than diffuse strong positivity; second, the diffuse strong positivity of CD10 may also support that the tumor component of alveolar adenoma is a type of monoclonal proliferation.

CD34 is known as a marker for hematopoietic stem and progenitor cells. It plays roles in various cellular processes including promoting proliferation, inhibiting differentiation, and influencing cell morphogenesis (12, 13). By reviewing relevant literature, we know that CD34, like CD10, possesses cellular stemness. CD34 promotes cell proliferation and differentiation. In the three cases we reported, CD34 was diffusely strongly positive in the stroma, and there is evidence to suggest that the stromal component of alveolar adenoma constitutes a neoplastic element. This inference is supported by observations analogous to those regarding CD10 expression: firstly, if the stromal component were non-neoplastic, CD34 expression would be expected to exhibit a patchy pattern; secondly, the widespread positivity of CD34 indicates that this stromal component may represent a monoclonal proliferative process.

As a classic anti-apoptotic factor, BCL-2 exerts a tumor-promoting effect (14). In the group of cases we reported, Bcl-2 showed focal positivity, along with diffuse positivity for CD10 and CD34. We speculate that the stromal component of alveolar adenoma may also be a tumor component.

Differential diagnosis: the differential diagnosis includes papillary adenoma, pulmonary hamartoma, sclerosing pneumocytoma, atypical adenomatous hyperplasia, lepidic adenocarcinoma, and lymphangioma (5, 15). Papillary adenomas are also benign lung tumors with clear boundaries, but they typically exhibit a characteristic branched, papillary architecture with a fibrous vascular core. In contrast, alveolar adenomas do not have a papillary structure, and their stroma is composed of spindle-shaped cells (16). Pulmonary sclerosing pneumocytoma (PSP) displays four morphological patterns: papillary, solid, vascular, and sclerotic. It consists of two cell types—interstitial cells express Vimentin and surface cuboidal cells express pan-CK, but both test positive for TTF-1. It is particularly noted that the stromal component of PSP expresses TTF-1 and EMA, whereas pulmonary adenoma does not express these markers (17). Lymphangioma resembles alveolar adenoma morphologically but can be distinguished by immunohistochemical expression of CD31, D2-40, and factor VIII, whereas the epithelium of alveolar adenoma expresses CK and TTF-1 (5).

During intraoperative frozen section analysis, it is crucial to differentiate alveolar adenoma from malignant tumors to prevent overtreatment. Helpful diagnostic clues include the presence of a well-defined nodule. Additionally, the epithelial lining of alveolar adenomas lacks atypia, mitotic figures, and necrosis. The stroma is rich in inflammatory cells such as lymphocytes and plasma cells, and eosinophilic secretions may be observed within cystic spaces.

Limitations: due to the limited number of cases, it is unknown whether CD34 and CD10 are continuously expressed in the stroma of alveolar adenoma. Since molecular testing was not performed, it is also unclear whether the stromal component of alveolar adenoma is neoplastic. We hope that future studies with more cases and molecular testing will help verify our hypothesis.

Conclusion: alveolar adenoma is very rare and this is the largest case analysis to date. This study found CD34 and CD10 positivity in stromal cells across all cases, which may aid in diagnosing alveolar adenoma. For the first time, a new perspective is proposed: that the stromal component may also be a part of the tumor. However, this is a cautious interpretation that requires further validation.

## Data Availability

The original contributions presented in the study are included in the article/supplementary material. Further inquiries can be directed to the corresponding authors.
